# The meaning of repeated assisted reproductive technologies failures experienced of older infertile women

**DOI:** 10.3389/frph.2025.1515086

**Published:** 2025-02-11

**Authors:** Hyun Jung Oh, Gisoo Shin

**Affiliations:** ^1^ The Graduate School of Nursing and Health, Chung-Ang University, Seoul, Republic of Korea; ^2^ College of Nursing, Chung-Ang University, Seoul, Republic of Korea

**Keywords:** infertility, women, treatment failure, reproductive techniques, assist, experiences

## Abstract

**Introduction:**

The trend of older pregnant women and infertile women in South Korea, who are influencing the low birth rate, is on the rise. Older infertile women earnestly hoped for pregnancy and persisted in undergoing assisted reproductive technology multiple times. However, contrary to their wishes, they experienced failure to conceive.

**Materials and method:**

This study is qualitative research aimed at exploring the essential meaning of the experiences of women over 35 who have faced pregnancy failure after assisted reproductive technology.

**Results:**

The average age of the participants in this study was 41.8 years, and they underwent an average of 5.7 assisted reproductive treatments after their infertility diagnosis. Through in-depth interviews with the participants, 120 meaningful statements were derived, which were classified into 23 themes, 9 theme clusters, and 4 categories. The four categories were “struggles and trials regarding assisted reproductive technology”, “the challenging treatment journey”, “the journey of overcoming sadness”, and “the value and happiness gained from experience”.

**Conclusion:**

The results of this study showed that infertile women over 35, despite failing to conceive after assisted reproductive technology, were able to overcome their sadness and move towards personal growth. Therefore, various methods should be sought to support the psychological growth of women undergoing assisted reproductive technology.

## Introduction

South Korea is one of the countries experiencing the most rapid decline in birth rates in the world, to the extent that concerns about population decline are turning into fears of national extinction. This decrease in birth rates is at a critically serious level globally, with government projections indicating that this low birth rate phenomenon will worsen. One of the factors influencing this low birth rate is infertility (1).

Infertility is defined as a reproductive system issue where a couple has not achieved pregnancy after a year of regular sexual activity (2). In South Korea, the number of infertility patients increased by 11.4%, and infertility is rising globally at a rate of 0.37% per year, with the worldwide prevalence of infertility estimated at about 15%. However, only about 3% of those affected are reported to be receiving treatment for infertility (3). The increase in infertility is attributed to several factors, including reduced reproductive ability due to older maternal age, an increase in reproductive disorders, frequent artificial abortions or natural miscarriages, increased use of excessive contraception for planned childbirth, stress, lack of exercise, and environmental pollution (4).

Currently, assisted reproductive technologies (ART), including *in vitro* fertilization (IVF), have shown positive effects in treating infertility and have been progressively advanced into high-tech solutions. ART encompasses all treatments and procedures related to handling eggs, sperm, and embryos for the purpose of achieving pregnancy (5). The world's first successful ART led to the birth of a healthy child through IVF in the UK in 1978, while in South Korea, the first test-tube baby was born via IVF at Seoul National University Hospital in 1985 (6). Types of ART include *in vitro* fertilization and embryo transfer (IVF-ET), intrauterine insemination (IUI), thawing embryo transfer (T-ET), and intracytoplasmic sperm injection (ICSI), often performed alongside ovulation induction to increase pregnancy rates (5, 6).

However, the success rates of ART remain relatively low. The diagnosis of infertility itself acts as a psychological stressor, accompanied by physical discomfort and economic burdens from medications and procedures for infertility treatment (7). Additionally, the expectations surrounding pregnancy success and the feelings of loss upon failure, as well as the stress from procedures, contribute to increased mental burdens. Infertility also brings about significant psychological changes, such as depression, social isolation, and lowered self-esteem in many women, with such psychological distress being reported as similar to that experienced by patients diagnosed with cancer or parents who have faced loss (8). Moreover, in South Korea, pregnancy and childbirth are not regarded as concepts that should be recognized and supported at a societal or national level. Instead, the responsibility is entirely placed on individuals and families, with the burden falling especially on women, who are the primary agents of pregnancy and childbirth. Women are held accountable and expected to fulfill their roles as reproducers. As a result, the suffering experienced by women who fail in ART is further intensified (9).

Despite this, infertile women often have no alternative treatment options and choose to undergo additional procedures following failed IVF attempts. The stress and burdens experienced in such situations tend to increase, and the repeated attempts at IVF can sometimes escalate into serious depressive disorders (10). Particularly, women over the age of 35 see their natural pregnancy rates drop to around 50%. For older women with infertility, the pregnancy rate from typical IVF procedures is approximately 25%, with a miscarriage rate of 30%–50% reported among them (11).

Currently, in South Korea, various personal and societal factors are contributing to a trend of delayed marriage and pregnancy (12). Older infertile women face psychological issues such as inferiority complexes, self-blame, lethargy, and loss of hope for the future when diagnosed with infertility, which can exacerbate the causes of infertility and create a vicious cycle (13). Therefore, to provide realistic support to older infertile women, it is essential to prioritize understanding their experiences and phenomena rather than classifying infertility and advanced maternal age strictly as medical conditions. Interventions and alternatives developed without a thorough understanding of older infertile women may have limited effectiveness. This study aims to explore the experiences and meanings of women over 35 who have undergone assisted reproductive technologies, providing foundational data for developing interventions and education programs to improve the quality of life for older infertile women.

## Materials and methods

### Research design

This study is qualitative research that applies a phenomenological approach through in-depth interviews to understand the experiences and meanings of women over 35 who have undergone ART.

### Participants

The participants in this study are 10 women aged 35 and older who failed to conceive after undergoing ART in Korea over the past 2 years. To recruit participants, we first obtained approval from the clinic's medical staff, then performed purposive sampling among infertile women visiting the clinic. After obtaining voluntary written consent regarding the purpose and methods of the study, interviews were scheduled at the times and locations desired by the participants.

### Data collection

Data collection was conducted through one-on-one in-depth interviews. The interview questions avoided structured survey formats and instead used semi-structured and open-ended questions, allowing participants to express themselves freely. The interview questions included topics such as the motivation to begin ART, the physical and psychological challenges or changes experienced during the procedure, the emotions felt after a failed pregnancy, the support or assistance received from others, and the personal significance of undergoing ART and coping with a failed pregnancy.

If additional explanations were needed during the responses or if relevant topics emerged, follow-up questions were asked to clarify those points. The interview questions began within a broad framework, and as the interviews progressed, context-appropriate questions were added to gain a more accurate and detailed understanding of the participants' inner experiences. Each interview lasted approximately 60 minutes, and each participant was interviewed 2–3 times. The interviews took place from May 4 to May 31, 2021. And the process was concluded upon reaching the saturation point, deemed sufficient based on Lincoln and Guba's criterion of information redundancy (14).

### Data analysis

Data analysis in this study followed Colaizzi's method (15). The research process is as follows. First, a research topic was selected to achieve the research objective, and participants were recruited, followed by data collection. The collected data was then reviewed to derive meaningful statements, and meanings were formed from these statements and their restatements. Subsequently, thematic clusters were categorized based on the formulated meanings, and the analyzed data was comprehensively explained according to the themes. The essential structure was clarified by integrating the common elements of the experienced phenomenon. Finally, the validity of the research findings was verified in collaboration with the research participants and qualitative research experts. The researchers endeavored to conduct the study in alignment with the evaluation criteria for qualitative research proposed by Lincoln and Guba (14). To ensure credibility, researchers aimed to interpret the phenomenon vividly and thoroughly by listening to the interview data immediately and reading it multiple times to extract its essential meanings. To enhance applicability, researchers discussed whether the findings represented unique experiences of the participants or could be more broadly applied. For consistency, researchers provided detailed descriptions of the research subjects, in-depth interview methods, procedures, and data collection processes, ensuring the study could be replicated in the future. Finally, to maintain neutrality, the researchers deliberately avoided conducting a literature review at the outset, worked to eliminate bias and suspend judgment during the process of extracting meanings, and emphasized maintaining an objective perspective on participants' experiences during discussions among the research team.

### Ethical considerations for research participants

Before conducting the study, approval was obtained from the Institutional Review Board (IRB No: 2021-03-033) of the hospital to which the researcher is affiliated. Participants were informed about the research purpose, duration, methods, and anticipated risks and benefits. They were assured that there would be no disadvantages for declining participation or withdrawing consent after agreeing to participate. Additionally, participants were informed that they could withdraw from the study at any time if they experienced psychological discomfort or stress during the interview process. All personal information of the study participants was protected by assigning serial numbers, and materials such as audio recordings, transcripts, and notes were stored on separate media. The researcher was aware that their personal conduct could cause discomfort or stress to participants and exercised caution in the use of terminology during interviews. Also, all participants in this study were given a small token of appreciation after the interview.

## Results

### Participant characteristics

The average age of the participants was 41.8 years, with an average infertility duration of 3.9 years and an average of 5.7 experiences with ART (Table 1).

**Table 1:** Characteristics of participants.

Participants	Age (years)	Duration of infertility (years)	Obstetric history^a^	Number of ART^b^
1	45	2	0-0-1-0	4
2	49	2	0-0-1-0	3
3	38	9	0-0-1-0	19
4	37	3	0-0-1-0	4
5	44	3	0-0-1-0	6
6	45	2	0-0-1-0	3
7	38	4	0-0-1-0	4
8	38	5	0-0-0-0	4
9	45	3	0-0-0-0	4
10	39	6	0-0-1-0	9

^a^ T-P-A-L, term birth-preterm birth-abortion-living baby.

^b^ ART, Assisted reproductive technology.

**Participant 1**: Married at 34, later in life, and lived without planning for children until her mid-40s, after 10 years of marriage. Following a visit to the emergency room for abnormal bleeding, she was advised to undergo a hysterectomy but was encouraged by her husband to preserve her uterus, leading her to start infertility treatment. She resigned from her job in January this year to focus on infertility treatment but is contemplating re-employment due to financial concerns.

**Participant 2**: After 10 years of dating, she married at 44, adhering to a non-marriage principle. Initially living as a DINK (double income, no kids) couple, she decided to plan for pregnancy at 48 after her father-in-law was diagnosed with lung cancer. Concerned about her impending menopause, she started preparing for pregnancy through ART but worries about being able to raise a child in her late 40 s.

**Participant 3**: Experienced early menopause after undergoing surgery for an ovarian teratoma at 23. She married at 30 and immediately began ART, attempting IVF over 19 times in 9 years. Despite repeated failures and the associated frustrations, she expressed her intention to try again soon.

**Participant 4**: Husband suggested having a child five years into their marriage, she underwent testing and received an infertility diagnosis, which shocked her. She quit her job to dedicate herself to ART. Although her first procedure resulted in an ectopic pregnancy and subsequent miscarriage, she succeeded after four more attempts and is expecting to give birth in mid-June.

**Participant 5**: Married at 30 without initially wanting children, she developed a desire for kids after undergoing a miscarriage due to an ectopic pregnancy four years ago. She blamed herself for starting late but felt that trying would prevent future regrets, prompting her to begin ART.

**Participant 6**: Attempted to conceive immediately after marrying at 41 but faced difficulties, prompting her to seek medical help. She was shocked to be diagnosed with infertility, endometriosis, and hypertension. Although her first implantation attempt succeeded, it ended in miscarriage, but she expressed determination to overcome the challenges for future attempts.

**Participant 7**: After three years of marriage without pregnancy, she sought help from a fertility clinic. Her husband expressed a strong desire to have children, even considering adoption, which led them to choose ART. She mentioned that her underlying conditions of diabetes and obesity made the stress of unsuccessful treatments particularly challenging.

**Participant 8**: Diagnosed with structural infertility at 24 after surgery for hydro-salpinx and endometriosis, she informed her husband about her infertility before their marriage at 34 and opted for ART without artificial insemination. She successfully conceived after just four procedures and is now awaiting a natural delivery.

**Participant 9:** Remarried at 42 after a divorce and diagnosed with infertility due to decreased ovarian function. She froze her eggs just before remarrying and, after the infertility diagnosis, attempted to use them in ART, but it was unsuccessful. Currently, she is undergoing ovulation induction and hopes for a miracle.

**Participant 10**: Diagnosed with infertility for both her and her husband after marrying at 32, she underwent six rounds of ART, all of which failed. Afterward, she tried natural conception at a natural pregnancy center, which also failed. At 39, she has given up on the hope of becoming pregnant and is now focused on caring for her dog, but she has no regrets about her attempts at ART.

### Analysis and categorization of participants' experiences

In this study, 120 meaningful statements were obtained through in-depth interviews with participants. From these, 23 themes, 9 clusters of themes, and 4 categories were derived and classified. The four categories ultimately classified, following the flow of experience, were: “struggles and trials regarding ART”, “challenging treatment journey”, “signs of positivity and hope”, and “value and happiness derived from experience”. The detailed structure of the theme clusters and themes for each category is shown in (Table 2).

1. Category 1: Struggles and trials regarding ART

**Table 2:** Categorization of the meanings related to experiences of failed assisted reproductive technology.

Categories	Clusters of themes	Themes
Struggles and trials regarding assisted reproductive technology	The inevitable choice of treatment	Becoming a patient with structural infertility
		Family demanding pregnancy and childbirth
		Ovarian reproductive age nearing menopause
	Lost in confusion right from the start	Financial difficulties leading to indecision
		Concerns about raising a child and anxiety about old age
		Lack of knowledge about infertility treatment
The challenging treatment journey	Emotional rollercoaster	Emotional ups and downs due to hormone therapy
		Expectation around embryo transfer
		Disappointment and frustration after implantation failure
	Unexpected trauma	Fear and anxiety from anesthesia awareness
		Depression from repeated treatment failures
	Persevering through hardship	Just one more time
		Wishing for pregnancy success
		Letting go
The journey of overcoming sadness	From sorrow to encouragement	The sadness of loss
		The journey of sadness
	The strength and comfort of shared support	A husband who thinks of me more than I do
		Genuine words build trust
		The unspoken power of empathy
The value and happiness gained from experience	The family became even more precious	The expectation of a family growing with children
		Reflecting on the meaning of family
	The strength to grow in life	I do my best to every process, not just the outcome
		An experience with no regrets

Participants expressed that while they were conflicted about deciding to undergo ART, they viewed it as an inevitable choice. At the same time, they also felt confused about the decision.

1.1. The inevitable choice of treatment

Most participants described their decision to start ART as an unavoidable choice due to their circumstances. Most of the participants were older women facing infertility, with ovarian function nearing its limits as they approached menopause. Some had structural infertility or felt pressured to choose ART after their husbands brought up the idea of adoption.

1.1.1. Becoming a patient with structural infertility

“That time, I had an ectopic pregnancy that ended in miscarriage and was treated in the emergency room, and that’s when it all started. I regained my emotional stability… (omitted) And right after that, I began IVF treatment.”<*Participant 5*>.

“Right after I got married… (omitted) I had a history of ovarian surgery, so I only did IVF from the beginning.”<*Participant 3*>.

1.1.2. Family demanding pregnancy and childbirth

“Actually, my husband wants a child because of my in-laws. But my own parents, well… they just say. As long as you two are happy, that's enough. Anyway, we think it will be difficult to have a baby because we are getting married late." <*Participant 2*>.

“My husband desperately wanted it… (omitted).”<*Participant 4*>.

1.1.3. Ovarian reproductive age nearing menopause

“I'm already at the age where I'm nearing menopause… But since I still have my period, I decided to try it this year, thinking it might be my last chance.” <*Participant 1*>.

“Given my age, the chances are low, so there was no other option.”<*Participant 6*>.

1.2. Lost in confusion right from the start

Even after making the difficult decision to start ART, participants constantly questioned whether it was the right choice. The biggest obstacle was financial concerns. Despite having saved up some money due to their age, they were anxious as their bank accounts kept depleting after each procedure, which cost tens to hundreds of thousands of won. Knowing that the treatment costs were a burden on their husbands, especially as the sole breadwinners, participants felt guilty and considered stopping the treatment to return to work. Another obstacle was their advanced age itself. Even if they had a child right away, they were worried about the physical limitations they might face when raising the child. Most participants also expressed a lack of knowledge about infertility treatments, describing themselves as older novices.

1.2.1. Financial difficulties leading to indecision

“My husband is happy and keeps telling me to continue, but honestly, I’m conflicted. I quit my job, so I want to get back into work… But it’s not easy to take time off at a new job, so deciding is hard for me. Meanwhile, my husband says to just rest and go to the hospital… But we’re struggling financially.”<*Participant 1*>.

“The truth is, this costs a lot of money, so I worry about that. I know it’s a financial burden on my husband…”<*Participant 4*>.

1.2.2. Concerns about raising a child and anxiety about old age

“On the one hand, I’m really worried. I’m so old that even having a child would be a miracle, and if I do have one, it might make the news… But even if I do, can I really raise the child? My body is also aging.”<*Participant 9*>.

“It’s not just about having a child at my age, but now we’d have to raise it. At this age, others are already getting ready to be grandparents…”<*Participant 2*>.

1.2.3. Lack of knowledge about infertility treatment

“Honestly… I've only recently started to understand what the doctors have been explaining. The injections I get seem slightly different each time… (omitted) In my case, I'm a novice. I didn't know anything… Everything is so unfamiliar.” <*Participant 10*>.

“During the first treatment, I didn’t know the order or anything. They’d just say, come in tomorrow at this time, and I’d just go and follow along without any real thought… Looking back, that time was really precious.”<*Participant 1*>.

“When I had to give myself the injections for the first time, I thought it would’ve been better if the nurses had shown me using the actual instructions and syringes as they explained it.”<*Participant 3*>.

2. Category 2: The challenging treatment journey

Participants expressed their experiences with ART as filled with a range of emotions they had never encountered in their life cycle before.

2.1. Emotional rollercoaster

Participants reported that when they began ART, they were administered hormone treatments, which led to both physical and emotional changes due to the hormones. They described it as experiencing menopause in a short time. Additionally, participants became emotionally attached to their embryos while waiting for the embryo transfer after egg retrieval, feeling excitement and hope. However, the disappointment and frustration felt after a failed embryo implantation were proportional to their high expectations, dragging their emotions down to the lowest depths.

2.1.1. Emotional ups and downs due to hormone therapy

“When you take hormone treatments, your emotions fluctuate. I could feel it, and so could my husband… Sometimes I’d burst into tears, and everything felt like the sky was covered in dark clouds… (omitted) I questioned whether this was the right thing.”<*Participant 10*>.

“I'd heard about the side effects of hormone treatments. I knew, but it was still hard when I was taking them. My emotional state became terrible.” <*Participant 4*>.

2.1.2. Expectation around embryo transfer

“Before the embryo transfer… I kept wondering, ‘What kind of child will come? Is it developing well?’ It felt like I had left my child there. I kept thinking, ‘Is it okay?’”<*Participant 8*>.

“During the waiting period for the embryo, I was always more hopeful. Whether it was the first, second, or third attempt, even with my current successful case, my expectations were higher than my anxiety.”<*Participant 5*>.

2.1.3. Disappointment and frustration after implantation failure

“When they told me no eggs had been retrieved, I felt like my heart completely collapsed. There are no words to describe it.”<*Participant 2*>.

“The frustration after a failure… I don’t want to go through that feeling again. Honestly… It feels like you hit rock bottom. Emotionally, it’s like… like you fall endlessly down to the bottom. It’s such an overwhelming feeling… and I thought, ‘I really don’t want to do this ever again.’”<*Participant 3*>.

2.2 Unexpected trauma

Participants considered whether to give up on the treatment due to emotions they had never experienced before, and some experienced anesthesia awareness due to resistance to the anesthesia used during the procedure. The vivid memories of the pain and discomfort experienced during the procedure led to fear and anxiety about undergoing future treatments. Moreover, the repeated failures accumulated into feelings of frustration, leading to depression, and the mental burden even led some to think about suicide.

2.2.1. Fear and anxiety from anesthesia awareness

“I’m not sure if it’s because I don’t respond well to anesthesia or if I’m just sensitive, but I could feel everything during the procedure. It was painful… So before the second or third egg retrieval, I was too scared to sleep, just constantly debating whether or not to go to the hospital.”<*Participant 4*>.

“I woke up from the anesthesia during the procedure and could hear the doctor talking and feel the pricking sensation. I remember all of it, and every time I had to undergo the procedure, I worried, ‘What if I wake up again today?’ I just hoped today wouldn’t be one of those days.”<*Participant 3*>.

2.2.2. Depression from repeated treatment failures

“Depression hit me as regularly as having a meal. Once, I was on the bus to the hospital, and I thought, ‘What if there’s an accident, and I die? Would I finally be free?’”<*Participant 10*>.

“I kept thinking, ‘Should I just give up on everything?’ I had that thought countless times. When I sank into the pit of depression, it took me months to swim out of it. I began to understand how people who commit suicide feel.” <*Participant 7*>.

2.3. Persevering through hardship

Despite the emotional rollercoaster and unexpected trauma, participants said they managed to pull themselves together again. This was because they believed it might be the last chance they would ever have, and they wanted to give their best effort to succeed in getting pregnant. At the same time, they were also practicing letting go of their attachment to the outcome of pregnancy success.

2.3.1. Just one more time

“Today is the youngest I’ll ever be. I felt that if I missed this opportunity, I might never get another chance.”<*Participant 10*>.

“Just one more time, just one last time… I was so desperate.”<*Participant 9*>.

2.3.2. Wishing for pregnancy success

“I really wanted to succeed in getting pregnant. I exercised, avoided using disposable containers to reduce exposure to environmental hormones… ”<*Participant 7*>.

“I ate foods that were supposed to be good for eggs, like tofu, soybeans, onion juice, and cabbage juice, desperately wishing for pregnancy success.”<*Participant 2*>.

2.3.3. Letting goes

“I was desperate, but I knew there was a chance of failure… Knowing that a part of me began to let go of the outcome.”<*Participant 9*>.

“They say that having a child is a gift from the heavens, that it’s not my fault. When I heard things like that, I’d think, ‘Maybe I’m just not meant to have a child in this life,’ and I would comfort myself with that thought.”<*Participant 6*>.

3. Category 3: The journey of overcoming sadness

Participants in this study experienced multiple instances of pregnancy failure following assisted reproductive treatments. They described the failure as an overwhelming sadness, akin to losing a loved one. Despite this, they reflected that the process of overcoming grief allowed them to discover inner strength and encourage themselves not to be defeated by sorrow. They also found support and comfort from their husbands and medical staff, which helped them gain strength in moments of sadness and despair.

3.1. From sorrow to encouragement

The participants said that the start of assisted reproductive treatments felt as if they had already received a pregnancy diagnosis, leading to emotional attachment. Because of these feelings, the process of implantation failure after the embryo transfer felt like a sadness caused by loss.

3.1.1. The sadness of loss

“Throughout the process of growing the eggs, *in vitro* fertilization, and implantation, I became emotionally attached as if I were raising a child. When the implantation failed, it felt like I had lost a baby, and the sense of loss and sadness hit me hard.”<*Participant 7*>.

“My heart sank. It was like hearing the news of a loved one’s death.”<*Participant 5*>.

3.1.2. The Journey of Sadness

“I can’t just let go of my dreams because I’m sad. I took the time to look within myself, to talk to myself about what I truly want… (omitted) Since this is a long journey, I must keep going.”<*Participant 6*>.

“Every time sadness overwhelmed me, I cheered myself on. You did well, you’re doing well, and you’ll continue to do well. You’re doing things that not everyone can do. I kept saying that to myself.”<*Participant 9*>.

3.2. The strength and comfort of shared support

Participants said that every time they went through assisted reproductive treatments and faced pregnancy failures, they felt like giving up on the journey to become a mother countless time. However, they also shared how the support and encouragement from their husbands and medical staff provided them with light amid their sadness and despair.

3.2.1. A husband who thinks of me more than I do

“The assisted reproductive process isn’t something I go through alone. My husband’s sperm is also collected at specific times. He had to take leave from work over ten times, and it must have been hard for him… but he always worried about my schedule and my physical condition.”<*Participant 3*>.

“He always comforted me when my emotions were all over the place, saying, it’s okay, this will pass.”<*Participant 10*>.

3.2.2. Genuine words build trust

“People going through assisted reproduction tend to believe anything they hear. There’s so much unverified information online. But they understood that and always gave me truthful information, even when I complained, which made me feel I could trust them with my body.”<*Participant 2*>.

“Trust is the most important thing for us. We go through the process of assisted reproduction not just once but several times, and without trust, it wouldn’t be possible.”<*Participant 1*>.

3.2.3. The unspoken power of empathy

“Honestly, if it weren’t for the doctors and nurses, I wouldn’t have been able to endure this tough process. They’re very busy, but they still took the time to look me in the eye, listen to me, and pat my shoulder. That simple act gave me so much strength.”<*Participant 4*>.

“Even on holidays and their days off, they came to the hospital to match my assisted reproduction schedule. I was so grateful. Every time I went in for a procedure, they held my hand..Lying on the operating table, in that unfamiliar and scary situation, I felt a moment of warmth”. <*Participant 8*>.

4. Category 4: The value and happiness gained from experience

The participants in this study underwent assisted reproductive treatments for periods ranging from 2 to 9 years, a considerable amount of time. Throughout these years, they expressed that they grew as individuals and found their experiences to be valuable from a life perspective.

4.1. The family became even more precious

Participants continued through the challenging process of assisted reproductive treatments because their ideal vision of a family was one where they grew alongside their children. However, as they repeated the treatments, they reflected on the true meaning of family.

4.1.1. The expectation of a family growing with children

“Looking around me, everyone has children within their family circle, so I naturally expected our family to grow with a child too”. <*Participant 3*>.

“I thought that a normal family meant growing with children, laughing and crying together.. I just wanted to have at least one child of my own”. <*Participant 7*>.

4.1.2. Reflecting on the meaning of family

“Before, I thought that once you got married, a family naturally followed. But after going through the unexpected process of assisted reproductive treatment, my bond with my spouse grew stronger, and I started thinking more about my parents.. I feel like my sense of family has deepened”. <*Participant 2*>.

“A family doesn't have to be connected by blood. If there are people who share meals and genuine feelings with me, I think they can grow old with me as my family. Of course, even a pet can be part of the family”. <*Participant 10>.*

4.3. The strength to grow in life

Though the process of assisted reproductive treatment was difficult and painful, participants felt it became a source of personal growth as women. Even if they could not fully fulfill the role of motherhood, it was an essential experience, and they had no lingering regrets.

4.2.1.1. I do my best to every process, not just the outcome

“I asked myself if women really must become mothers. Even if I couldn't become a mother, I told myself to try and give my best.. I think that was a step forward in my growth as a woman”. <*Participant 5*>.

“I imagined various possible futures. But the outcome of assisted reproductive treatment isn't something I can control. I don't know what situation or outcome will arise, but if I give my best in every moment with a clear direction, I believe I'll make the right choices in the future”. <*Participant 1*>.

4.2.2. An experience with no regrets

“At this stage of my life, it was the right choice to make. Regardless of the result, it was the most meaningful and regret-free experience of my life”. <*Participant 9*>.

“I don't think any experience is wasted. Through my experience, I can help other women struggling with infertility, or I can offer positive words of psychological support to families having difficulty raising children. That's why I believe it was a necessary experience”. <*Participant 6*>.

## Discussion

This study aimed to understand the experiences and meanings of ART among older infertile women in South Korea. The findings were structured into four categories, which will be discussed in detail.

The first category is `concerns and trials regarding the procedure'. This background explains why participants began ART. They reported that, due to physical changes associated with aging and societal pressures from family, they felt compelled to choose ART. Women's reproductive capabilities significantly decline after age 35, and with the increasing marriage age in South Korea, the number of older infertile women is also rising (3). According to 2023 statistics, the average marriage age for South Korean women was 33.6 years, with reasons for delayed marriage including the burden of balancing work and childcare, concerns about career interruptions, and financial pressures (1, 6). The declining marriage rate and rising marriage age are not unique to South Korea but reflect a global trend, indicating changes in societal and family structures (16). Despite these changes, social norms persist in South Korean families, often pressuring women regarding pregnancy decisions (9, 17). Infertile women undergoing ART are generally aware that the process involves not only the side effects of ovulation-inducing drugs but also various physical and mental hardships (18). Nonetheless, the choice to pursue ART is often intertwined with a patriarchal culture that views childbirth as an obligation for married women (19). Therefore, it is essential to consider both age-related physical changes and the social pressures that can disrupt a woman's self-esteem when making decisions about ART. This is because studies worldwide have shown that similar experiences occur regardless of culture (20).

The second category, “the challenging journey of procedures”, describes the experiences participants faced during the ART process. They endured numerous efforts and pains to realize their dreams of motherhood. Most women reported emotional fluctuations, experiencing joy and sorrow due to the high doses of hormones received at the first stage of ART. Hormone therapy is crucial for egg maturation, retrieval, and thickening the uterine lining, yet it can also induce anxiety and depression, making women sensitive to minor issues or easily fatigued (21). Moreover, the success of egg retrieval and embryo transfer brings great hope for pregnancy, but a failed implantation can lead to profound despair, creating a rollercoaster of emotions. Because the overwhelming fear is the feeling that they may never be able to conceive (22). Therefore, repeated failures in ART attempts and pregnancy have been reported to lead to depression and even suicidal thoughts among infertile women (23). This has been identified as a significant reason for discontinuing ART. Depression, anxiety, and stress resulting from ART experiences are correlated with quality of life and can negatively affect marital relationships (24). Infertile women are informed that the success rate of ART is only about 25%–35% (3–5), which reinforces the notion of their efforts. Consequently, these women actively seek and share knowledge related to ART both online and offline, trying various methods to achieve pregnancy (25). Participants expressed a mix of letting go of their expectations while clinging to the hope of one last attempt to succeed. Therefore, it is vital to monitor and counsel the emotional changes of women undergoing repeated ART, providing immediate interventions in consultation with professionals when necessary.

The third category is “the journey of overcoming sadness”. This reflects the emotions of sadness experienced by participants during ART and failed embryo implantation, along with the significance of support systems from family and medical staff. From the beginning of ART, infertile women perceive embryos as having the potential for life and imagine them growing into children. Consequently, experiencing failed implantation brings the grief of losing a child, which can negatively impact their reproductive identity. However, these women also express their determination to overcome sadness and endure all necessary efforts to achieve motherhood. ART is seen as a process with stages, with failed implantation viewed as a significant obstacle to be overcome (22). The support from husbands and medical professionals plays a crucial role during this grieving process. Previous research indicates that the partner has the most significant impact during ART, reducing stress related to the procedure and the depression stemming from failed implantation (24, 26). Additionally, empathy and support from medical staff positively influence infertile women, encouraging active participation in their treatment (27). Women undergoing ART desire to be seen as individuals rather than merely subjects of technical procedures, emphasizing the need for emotional exchanges that acknowledge their pain. Thus, it is essential for partners and medical professionals to seek supportive measures throughout the ART process.

Finally, the fourth category discusses the value and happiness derived from experiences. Women undergoing ART often experience existential crises and negative impacts on occupational balance because of embryo failure (28). However, women undergoing ART for an extended period often view resilience as a coping strategy, using these experiences as an opportunity to reflect on their lives and redefine their paths (29). Resilience refers to the positive strength and capabilities individuals possess to overcome difficulties or stress, interpreted as the ability to cope effectively in frustrating or crisis situations (30). This resilience can be reinforced not only by the individual but also through support from partners, family, and those around them, positively influencing the well-being and growth of women facing stigmatization or chronic health issues (31). Growth involves changes in one's perspective on life, interpersonal relationships, and philosophical understandings. As a result, participants reflected on their experiences and returned to their families, forming new family relationships, which involve changes not only in thought processes but also in networks of gender, work, community, and intimacy (32). This outcome signifies liberation from the societal chains that dictate that women must become mothers, allowing them to escape feelings of guilt and inferiority tied to being perceived only as mothers. Therefore, it is crucial to deeply understand the isolated triggers experienced by infertile women who undergo ART and support the positive transformation of their self-image.

## Conclusion

This study analyzed the experiences of infertile women who faced pregnancy failure after ART. It found that they chose ART as an unavoidable option and endured the challenging process with hope and anticipation. Despite experiencing sadness after failed pregnancies, they managed to overcome this sorrow with the support of their partners and medical staff, leading to personal growth. However, since this research was based on data obtained from a small number of interview participants at a specific infertility clinic in South Korea, the objectivity may be somewhat lacking. Additionally, since the research results are not derived from a diverse group of participants, there are limitations in generalizing the findings to all women who have failed to conceive after ART procedures. Despite these limitations, the study provides valuable qualitative insights into the meanings of ART experiences. Future research should include a broader range of cases involving various characteristics of women undergoing ART and explore interventions to support the psychological growth of these women.

## Data Availability

The original contributions presented in the study are included in the article/Supplementary Material, further inquiries can be directed to the corresponding author.
