# Supplementary material for: The meaning of repeated assisted reproductive technologies failures experienced of older infertile women
Source: Front Reprod Health. 2025 Feb 11;7:1515086. doi: 10.3389/frph.2025.1515086 (PMC11850572; doi:10.3389/frph.2025.1515086)
Supplement: Supplementary file 1 [file Datasheet1.pdf]

통지서

※ 본 과제의 문서보존기간은 3 년입니다.					
수신 - 의뢰(지원)기관	내부과제				
수신 - 연구책임자	산부인과 외래 오현정				
IRB File No.	ISPAIK 2021-03-033-002	심사내용	시정계획서	통지일자	2021.05.25
연구과제명 - 국문	고령 난임 여성들의 보조생식술 경험과 의미				
연구과제명 - 영문	The Experiences and Meanings of Elderly Infertile Women Undergoing Assisted Reproductive Technologies.				
임상시험코드		Study Nick Name			

연구분류1	☐ 약물 ☐ 생물학적 제재 ☐ 세포치료제 ☐ 건강기능식품
	☐ 의료시술 ☐ 의료기기 (○ 1등급 ○ 2등급 ○ 3등급 ○ 4등급)
	■ 해당사항없음
연구분류2	■ 인간대상연구 ☐ 인체유래물(검체)연구 ☐ 의무기록연구
	☐ 유전자연구 ☐ 유전자치료
	☐ 배아연구 ☐ 체세포복제배아연구 ☐ 줄기세포주연구
	☐ 기타 ( )
연구분류3	◉ 전향적 연구 ○ 후향적 연구 ○ 전향적 & 후향적 병행연구
연구분류 4	☐ 중재연구 ☐ 설문조사 ☐ 자료분석 및 분석연구
	☐ 관찰연구 (☐ 단면조사연구 ☐ 환자대조군연구 ☐ 코호트 연구)
	■ 기타 (질적연구)
연구분류 5	☐ 인간을 대상으로 하지 않는 연구 Non-clinical study (in vitro. in vivo preclinical study)

일반명		상품명			
전체피험자증례수	전체	6-10 명	국내	6-10 명	본원 6-10 명
연구승인기간	2021.05.17 ~ 2022.05.16				

본 서식은 전자서식(PDF 파일)으로 발급되었습니다.

바코드가 입력되지 않은 전자서식은 확인용 전용뷰어로 진본 여부를 확인할 수 없으며, 진본 여부가 표시되지 않습니다.

<table>
<tr><td>지원의뢰기관</td><td>기관명</td><td>내부과제</td><td>대표(직위)</td><td></td><td>성명</td><td></td></tr>
<tr><td>제출서류목록</td><td colspan="6">(첨부) 4. 연구진 업무 위임기록지(필수) [2.1] []
(첨부) 7. 피험자 설명문 및 동의서 [1.0] []
(첨부) 변경대비표 [2.0] []</td></tr>
<tr><td>관련근거</td><td>평가일자</td><td colspan="5">2021.05.17</td></tr>
<tr><td>중간보고시기</td><td colspan="2">2022년 05월 16일</td><td>비고</td><td colspan="3"></td></tr>
<tr><td>심사결과</td><td colspan="6">◉ 승인 ○ 시정승인</td></tr>
<tr><td>심사결과</td><td colspan="6">[이전(2021.4.13)지적사항]
1)연구계획서 와 동의서 간, 피험자 모집 명수 차이
(6-10명 vs 10명)가 있어 일치필요
2)연구진 업무 위임기록지:2,8 업무의 배정이 필요함.
2. 선정 / 제외기준 최종 확인(의사만 해당)
8. 증례기록서 최종 확인(PI만 해당)
3)신청서2: 모집방법:
원내광고를 할 예정인지 명확하지 않습니다.
광고 부분 추가된다면 광고문 제출이 필요합니다.

이전 지적 사항에 대해 보완이 이루어져 승인합니다.
※ 동의서 다운방법: eIRB 시스템 로그인 후 좌측의 [동의서 인증] → [다운로드]를 선택 후 출력하여 인증된 동의서로 사용해 주시기 바랍니다.</td></tr>
</table>

[연구자 보고 의무사항]
(1) 중간보고서(지속심의) 제출: 연구계획의 승인유효기간 만료 2개월 이전에 중간보고서를 제출해주십시오.
(2) 종료보고서 제출: 종료보고서는 연구 종료예정일로부터 2개월 이전에 제출되어야 합니다. 본 원 피험자의 등록이 종료되고 약물투여 등의 처치가 완료되는 시점에서 종료보고서를 제출해 주십시오.
(3) 결과보고서 제출: 연구결과가 모두 수집되고 이에 대한 평가가 완료된 시점에서 결과보고서를 제출해 주십시오.
(4) 연구 중 중대한 이상반응 발생시 연구책임자는 본 위원회에 즉시 보고해야 합니다.

[연구자 준수사항]
* 연구책임자는 심의 결과에 이의가 있는 경우 위원회 통보일로부터 6개월 이내에 온라인으로 이의 사유를 기록하여 이의를 제기할 수 있습니다. 단, 동일 사항에 대하여 2회 연속으로 이의 신청을 할 수 없습니다.
* 책임연구자는 위원회의 승인(도장)을 받은 피험자 동의서만 사용할 수 있습니다.
* 강제 혹은 부당한 영향이 없는 상태에서 충분한 설명에 근거한 동의 과정을 수행할 것이며, 잠재적인 피험자에게 연구에의 참여여부를 고려할 수 있도록 충분히 기회를 제공해야 합니다.
* 피험자 모집광고는 사용 전에 위원회로부터 승인을 받아야 합니다.
* 연구자는 임상시험계획서 및 변경계획서의 승인 이전에 피험자의 해당 임상시험 참여 금지해야 합니다.
* 연구자는 변경계획서에 대한 승인 이전에 원 임상시험계획서와 다른 임상시험 실시를 금지해야 합니다(단, 피험

본 서식은 전자서식(PDF 파일)으로 발급되었습니다.

바코드가 입력되지 않은 전자서식은 확인용 전용뷰어로 진본 여부를 확인할 수 없으며, 진본 여부가 표시되지 않습니다.

자의 즉각적 위험요소 제거와 사소한 변경은 제외합니다).
* 본 위원회는 국제 임상시험 통일안 (ICH) 및 임상시험관리기준(GCP)을 준수합니다.
* 임상시험실시기관의 사전 서면동의 없이는 학술 이외의 목적으로 실시 기관명을 명시한 임상연구결과를 이용할 수 없습니다.
* 본 통지서는 KGCP 제 13조 ①항에 따른 심사통보서로 사용할 수 있습니다.
* 1, 2, 3상 임상연구는 의학연구윤리심의위원회의 승인과 함께 식품의약품안전처 승인을 얻은 후에 실시할 수 있습니다.
* 헬싱키 선언에 따라 모든 임상시험은 첫 피험자 모집하기 전 공개적으로 접근이 가능한 임상연구등록시스템 (Primary Registry)에 등록하여 이를 공개하여야 하며, 예를 들어, 질병관리본부에서 운영하는 임상연구정보서비스 (CRiS,http ; / / cris.cdc.go.kr)를 이용하실 수 있습니다.

인제대학교 일산백병원 의학연구윤리심의위원회위원장

일산백병원의
학연구윤리심
의위원회위원장

본 서식은 전자서식(PDF 파일)으로 발급되었습니다.

바코드가 입력되지 않은 전자서식은 확인용 전용뷰어로 진본 여부를 확인할 수 없으며, 진본 여부가 표시되지 않습니다.
