# Supplementary material for: The meaning of repeated assisted reproductive technologies failures experienced of older infertile women
Source: Front Reprod Health. 2025 Feb 11;7:1515086. doi: 10.3389/frph.2025.1515086 (PMC11850572; doi:10.3389/frph.2025.1515086)
Supplement: Supplementary file 2 [file Datasheet2.pdf]

인제대학교일산백병원
INJE UNIVERSITY ILSAN PAIK HOSPITAL
인제대학교 일산백병원 IRB APPROVED 2021. 05. 17

연구대상자 동의서

연구제목	고령 난임 여성들의 보조생식술 경험과 의미
※ 아래항목에 대해 동의한다면 해당부분에 √ 표기하시기 바랍니다.	
☑	본인은 이 설명문을 읽었으며, 본 연구의 목적, 방법, 기대효과, 가능한 위험성, 타 치료 방법의 유무 및 내용, 건강 정보 관리 등에 대한 충분한 설명을 듣고 이해하였습니다.
☑	모든 궁금한 사항에 대해 질문하였고, 충분한 답변을 들었습니다.
☑	본 연구에 동의한 경우라도 언제든지 철회할 수 있고, 철회 이후 다른 적절한 치료를 받을 수 있음을 확인하였습니다.
☑	이 연구목적으로 개인(민감)정보 수집· 이용· 제공 등에 관한 설명을 이해하였습니다.
☑	본인은 설명문 및 작성된 동의서 사본 1 부를 받았음을 확인합니다.
☑	충분한 시간을 갖고 생각하였으며, 본인은 이 연구에 참여하기를 자유로운 의사에 따라 동의합니다.

※ 각 관계자 1 명만 기입하되 만 19 세 미만의 미성년의 경우 본인을 포함 법정대리인 서명 필요

구분	내용
**연구대상자 서명**	성명: 이수지 (서명 또는 날인) [illegible]
	서명일: 2021 년 5 월 10 일
**연구대상자 대리인 서명** **(친권자, 배우자, 또는 후견인)** ** 해당하는 경우*	성명: (서명 또는 날인)
	대상자와의 관계:
	서명일: 년 월 일
**참관인 서명** **(시험자/대상자/대상자와의 대리인과는 무관한 제 3 자여야 합니다.)** ** 해당하는 경우*	성명: (서명 또는 날인)
	대상자와의 관계: (신분, 입회 사유 등)
	서명일: 년 월 일
**연구책임자/ 공동연구자 서명**	성명: [illegible] (서명 또는 날인) [illegible]
	서명일: 2021 년 5 월 10 일

본 동의서는 의학연구윤리심의위원회(IRB)에서 심의하여 사용을 승인한 동의서로, 일산백병원 IRB 직인이 찍힌 경우에만 유효합니다
